# Impact of Silver-Coated Dressing on Adipose-Derived Stem Cells and Fibroblasts in 2D and 3D Cultures

**DOI:** 10.3390/biomedicines14040776

**Published:** 2026-03-29

**Authors:** Alina Chelmuș-Burlacu, Eric Tang, Snejana Smolenschi-Palanciuc, Vlad Pieptu, Dragoș Pieptu

**Affiliations:** 1 Department of Plastic and Reconstructive Surgery, Grigore T. Popa University of Medicine and Pharmacy Iasi, 700115 Iași, Romania; smolenschi.snejana@gmail.com (S.S.-P.); dr.pieptu@gmail.com (V.P.); dragos.pieptu@umfiasi.ro (D.P.); 2 Aristan Laboratories Ltd., Macclesfield SK10 3HZ, UK; eric.tang@aristanuk.com; 3 Plastic Surgery Department, Regional Institute of Oncology, 700483 Iași, Romania

**Keywords:** silver-coated dressing, adipose-derived stem cells, fibroblasts

## Abstract

**Background/Objectives:** The effects of silver-coated dressing on wound healing, including cytotoxicity, are controversial due to the limited and incongruous results of in vitro versus in vivo research. Multiple factors intervene in wound healing processes and scarring, including pro/anti-inflammatory and pro/anti-fibrosis markers. Herein, to elucidate reported differences between in vitro and in vivo results, the effects of silver-coated dressing on 2D and 3D mono- and cocultures of fibroblasts and adipose-derived stem cells (ADSC) were investigated. **Methods**: Migration profiles in 2D and 3D assays, α-smooth muscle actin and proliferation marker Ki-67 expression, TGF-β1, TGF-β3, IL-6 and IL-10 levels and/or gene expression were assessed on four culture constructs. **Results**: In 2D systems at 24 h, silver-treated ADSC monocultures displayed better migration abilities compared to cocultures with high fibroblast ratio. In contrast, changes in the sprouting pattern between treated and untreated samples were non-significant in 3D constructs. TGFβ-1 levels decreased post-treatment, while TGFβ-3 increased, especially in 3D models. IL-6 gene expression was up-regulated following silver exposure in 3D models, mainly for stem cells in mono- and cocultures. **Conclusions**: Experiment data on 3D constructs suggest that silver-coated dressings do not significant impede wound healing, whereas cytotoxic effects were more pronounced in the 2D cultures. These inconsistencies, also noted in the literature, invite a methodological discussion of the 2D setup implications, recommending 3D constructs as a more appropriate evaluation standard where observable effects are closer to in vivo conditions and more relevant for transfer to clinical applications.

## 1. Introduction

In vivo, wound healing is a complex process with three overlapping phases: inflammation, proliferation, and remodeling [1,2]. Some researchers also consider hemostasis as a separate process, as wound repair cannot be initiated without it. Various factors can impact the physiology of wound healing, leading to delayed healing and chronic wounds, or the formation of abundant scar tissue [3]. The transforming growth factor beta (TGF-β) family of multifunctional peptides play a crucial role in all wound healing phases, modulating cell migration and differentiation, extracellular matrix synthesis and remodeling, and pro-/anti-fibrotic balance [4,5,6,7]. The isoforms TGFβ-1 and TGFβ-3, specifically, are known for their role in scar tissue formation [8].

Inflammation is vital for optimal wound healing, and any imbalance between pro and anti-inflammatory factors can lead to impairment [3,4,9]. For instance, the pro-inflammatory effects of pleiotropic cytokine interleukin 6 (IL-6) are well documented, although IL-6 has recently been shown to also exert anti-inflammatory and pro-regenerative effects, depending on the intracellular signaling pathway. Out-of-range IL-6 secretion can delay epithelization or overstimulate fibroblasts and, by consequence, scar formation [3,10,11,12]. An important anti-inflammatory counterpart is the cytokine interleukin 10 (IL-10), with known roles in modulating inflammation and extracellular matrix formation during wound healing [4,13,14,15].

Moreover, the regenerative potential of a wound is determined by the quantity and quality of the dermo-epidermal junction, the presence of stem cells in the wound and the ability to further recruit stem cells from other organs, such as chemotaxis from the bone marrow [16,17]. Stem cell homing to the wound, with subsequent differentiation and synthesis of pro-regenerative, angiogenic, and immunomodulator factors, can accelerate healing and reduce scar tissue formation [16,18,19,20]. In fact, stem cell therapy has slowly emerged as a useful accelerator in patients with low wound healing potential; at present, novel dressings and skin substitutes based on stem cells and/or their associated growth factors are under clinical development [16,18,21].

To further promote healing, topical antimicrobial agents are required to control the wound bioburden [2,22]. Silver is one of the most commonly used topical antiseptics for chronic wounds and burns. A new generation of silver-coated dressings (Ag), such as Acticoat^TM^, are now available to overcome the limitations of conventional silver nitrate and silver sulfadiazine treatments [22,23]. However, the effects of silver-based treatment in terms of cytotoxicity and promoting or delaying wound healing are controversial. Several in vitro studies previously suggested that silver may have a detrimental cytotoxic effect on keratinocytes, fibroblasts, and stem cells, while other studies found silver-based treatment to support wound healing in in vivo models [22,24,25]. Conventionally, previous in vitro studies on keratinocytes and fibroblasts are mainly performed as 2D cell cultures, which do not fully reflect the physiology of in vivo environments. Both in vitro and in vivo research is needed to establish the effects of silver-based treatment on stem cells in wound healing [26,27]. Nevertheless, major advances in cell culture research and a growing number of related studies have recently demonstrated that 3D in vitro cellular models are better at mimicking in vivo physiology than conventional 2D cultures; this is likely due to similarities with in vivo microenvironments and presents the added advantage of narrowing the gap to research on animal models [28,29].

The different benefits and implications of using silver nanoparticles in would healing are still being evaluated and discussed in the literature. In one in vivo study, a scaffold of silver nanoparticle-loaded collagen/chitosan was found to have anti-inflammatory effects and promote wound healing in vivo [30]. Another in vitro study reported cytotoxic effects of silver nanoparticles on stem cells [26].

To the best of our knowledge, the evidence is yet limited regarding the concurrent use of stem cells and silver-coated dressings, and their joint impact on cytokine balance. With this study, our aim is to contribute to the understanding of the effects that silver dressing can exert on cellular processes and constituents in wound healing, while also corroborating and comparing available in vitro methodological options. Specifically, we assessed migration potential, proliferation, dynamic cellular interaction and cytokine levels, and/or gene expression.

## 2. Materials and Methods

This was an in vitro study where 2D and 3D culture models were employed, and the effects on monotypic cultures and cocultures of key cell types involved in wound healing were compared. The coculture model constructs includes the normal human dermal fibroblasts (NHDF) and adipose-derived stem cells (ADSC); cocultures were prepared in two different ratios to reflect the heterogeneity of different microenvironment scenarios. Stem cell population may become a major fraction by intrinsic chemotaxis, external engraftment, or exogenous augmentation in stem cell therapy scenarios.

### 2.1. Cell Lines and Transfection

The two primary cell types used were normal human dermal fibroblasts (NHDF, C-12302 Promocell, Heidelberg, Germany) and human adipose-derived stem cells (ADSC, PT-5006, Lonza, Basel, Switzerland). The monocultures were grown in specific cell culture media: FGM-2 Bullet Kit (CC-3132, Lonza) for fibroblasts, ADSC^TM^ Growth Medium (PT-4505, Lonza) for stem cells, and a 1:1 mixture for the cocultures. All cells were cultured according to the manufacturer’s instructions. To distinguish and track the two different cell types in the cocultures, each cell type was genetically modified to express a different fluorescent protein. Each cell type was plated and expanded in 75 mL culture flasks for two passages, then the cells were detached with TripLE trypsin (12604013, ThermoFisher, Waltham, MA, USA), collected, and washed with PBS (14040133, ThermoFisher). They were further spun down and plated in 24-well plates to be transfected with 3rd generation lentivirus particles containing genetic insert coded for the corresponding fluorescent protein. The ADSC transfection was performed using GFP (EF1a)-Puro (LVP426, Amsbio, Abingdon, UK) in order to express the green fluorescent protein with emission at 525 nm. For NHDF, RFP (EF1a)-Puro (LVP429, Amsbio) red fluorescent protein expression with emission at 620 nm was used. The recommendations of the lentivirus manufacturer were followed for the transfection, while the selection of the transfected cells was performed accordingly to the kill curve. Prior to using the cells in any of the experiments, the p24 protein was assessed in the cell supernatant using ELISA (Lenti-XTM p24 Rapid Titer Kit; 63220, Clontech, Mountain View, CA, USA) to confirm safe usage with no measurable presence of virus particles in the resulting cultures. Cells were maintained and used up to passage 8 for all the studies. In this report, the wound healing response and effects of silver ions were studied in four different culture scenarios: stem cells and fibroblasts monocultures (ADSC and NHDF) and their cocultures at cell type ratios of 4:1 and 1:4, respectively (referred to below as NHDF:ADSC 4:1, A20N80; NHDF:ADSC 1:4, A80N20).

### 2.2. Silver-Coated Dressing

The silver-coated dressing (Ag) used in this study was the 5 × 5 cm three-layer dressing Acticoat^TM^ (6600808, Smith&Nephew, Watford, UK) containing 21–33.5 mg silver/dressing. In aqueous solution, silver is rapidly released to a steady state dissolution of 70–100 mg/L. To expose the cell cultures to it, the dressing was cut under sterile conditions into 5/5 mm fragments containing approximately 0.21–0.33 mg/mm^2^ of silver [22,23,31]. The working volumes used to expose the cell cultures to silver-coated dressing in this study were 500 µL for 2D cultures (amounting to a total dose of 40 µg of silver) and 200 µL for 3D cultures (16 µg). Although the total dose between 2D and 3D setups was different due to the working volumes, the difference was compensated for by varying the exposure times, as described below.

### 2.3. Wound Healing Assay—Cell Exclusion Zone Assay

**Figure 1:**
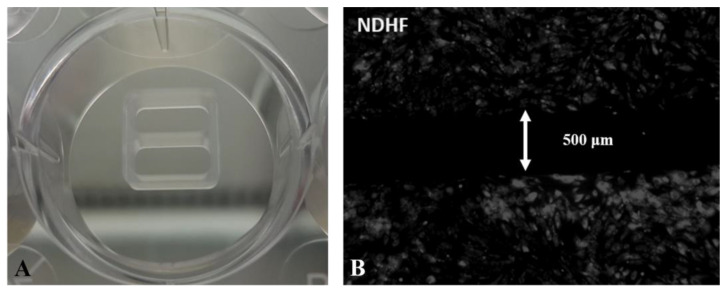
Cell exclusion zone assay: (**A**). Ibidi insert in a well (24-well plate); (**B**). NHDF monoculture after removing the Ibidi insert, with a defined gap of 500 µm, before treatment, 5× magnification.

**Figure 2:**
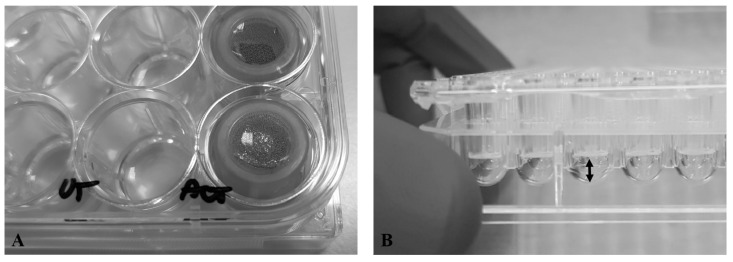
Experiment design for silver-coated dressing exposure: (**A**). 2D cultures in a 24-well plate-cropped snap cap with a 35 µm nylon mesh from a flow cytometry test tube in a well; (**B**). 3D cultures—a Millicell-96 Cell Cultured Insert Plate adapted to a 96-ULA plate, offering sufficient space (black arrow) for spheroid development.

To evaluate wound healing, a cell exclusion zone assay was performed using Ibidi insert (80209, Ibidi GmbH, Gräfelfing, Germany), which provides two cell culture compartments separated by a 500 µm thick wall [32,33]. The inserts were placed in 24-well plates (CLS3527-100EA, Sigma-Aldrich, Saint Louis, MO, USA), with one insert per well (Figure 1A). Following cell detachment with TripLE trypsin (12604013, ThermoFisher), viability was assessed with Trypan Blue (T10282, ThermoFisher) and cells were counted by an automated system (Countess Automated Cell Counter, ThermoFisher Scientific Invitrogen). Next, reservoirs of the Ibidi insert were seeded with a cell suspension of 7 × 10^4^ cells in 70 µL media per each cell condition and incubated for 12 h at 37 °C, 5% CO_2_. A volume of 500 µL of the corresponding medium was added to each well, outside the Ibidi insert, to preserve cell sheet integrity when the device was removed (Figure 1B). Then, the cultures featuring all four studied conditions were exposed to the silver-coated dressing fragments, suspended in cut flow cytometer caps plunged in the wells, with no contact with the bottom cell layer, and incubated for 24 h (Figure 2A,B). Cultures were then washed twice with warm PBS (14040133, ThermoFisher) and incubated at 37 °C, 5% CO_2_. After 24 h of exposure, wells were imaged by a Leica C6800 fluorescent microscope with image acquisition system (Leica Microsystems, Wetzlar, Germany).

### 2.4. Collagen Sprouting Assay

Spheroids were generated in 96 U-bottom well plates with ultra-low attachment (650979, Greiner, Kremsmünster, Austria) for all four cell modeling conditions, using the method described by Ivascu and Kubbies [34]. Cells from sub-confluent monolayer cultures were detached from flasks, counted, and suspended in cold culture medium (4 °C) with 1% Matrigel (356237, Corning, Corning, NY, USA). For each condition, 2 × 10^3^ cells were seeded in 200 µL medium/well; for cocultures, we used the same ratios as for 2D cultures. The plates were centrifuged at 300× *g* and 4 °C for 5 min, and then incubated for 3 days at 37 °C, 5% CO_2_, humidified atmosphere. To expose the spheroids to silver-coated dressing fragments, we used a Millicell-96 Cell Cultured Insert Plate (PSH004S5, Millipore, Burlington, MA, USA) adapted to a 96-ULA plate (Figure 2B). This setup allowed for contactless 72 h exposure of the spheroids to the silver-coated dressing fragments through a 0.4 µm filter. Additionally, the filter retained any debris from fragments, enabling imaging assessment.

For spheroid sprouting assay, we first coated 24 well plates with collagen type I (50201, Ibidi GmbH) by adding 200 µL/well of 5 mg/mL collagen at 4 °C; we then stood the plates on ice for 5 min. Each spheroid was transferred into the assay plate in 10 µL cell culture medium together with 60 µL of collagen mix, and transferred into each well of the coated 24-well plate. The spheroids were dispensed in droplets from a mix of collagen, M199 medium (M0650, Sigma-Aldrich) with 1% Glutamax (35050061, ThermoFisher), and 7.5% sodium bicarbonate (S8761, Sigma-Aldrich). Plates were incubated for 5–10 min at 37 °C, 5% CO_2_, humidified atmosphere, to allow the gelation of collagen; then, 500 µL of warm culture medium was added, followed by 3-day incubation at 37 °C, 5% CO_2_, humidified atmosphere. Once sprouting was observed via phase contrast microcopy, image acquisition was performed using fluorescent microscopy (Leica C6800, Leica Microsystems) with corresponding filters for each cell labeling.

To proof spheroid viability following the collagen sprouting assay, a viability test was performed using SytoxBlue 5 mM in DMSO (S11348, Thermofisher) at 1:2500 final dilution. A total of 20 µL solution was added over the collagen embedded spheroids and incubated at room temperature for 1 h, followed by image acquisition with Leica CM6800. Additionally, to evaluate the spatial arrangement of the cocultures, confocal images were acquired for the untreated conditions.

### 2.5. Immunofluorescence

Specific protein expression was evaluated by immunofluorescence. Primary antibodies dilutions were of 1/500 concentration for α-SMA (ab32575, Abcam, Cambridge, UK) and 1 µg/mL for Ki-67 (ab92742, Abcam). The secondary antibody (A32733, ThermoFisher) was used in 1/500 concentration, conjugated with Alexa Fluor Plus 647 (far-red). Nuclear counterstaining was performed using a 10 mg/mL Hoechst solution (H3570, ThermoFisher) at 1/5000 dilution.

After pre-incubation of the 2D setup for 24 h and 72 h for the 3D cultures, the silver dressing fragments were removed and the cultures were incubated for another 5 days at 37 °C in humidified atmosphere, 5% CO_2_. Subsequently, all cell cultures were washed twice in PBS and fixed with 4% paraformaldehyde solution for 40 min. Following 3 more PBS washes, the 2D-culture plates were incubated for 30 min and the 3D cultures for 1 h, with blocking buffer made of 0.3% bovine serum albumin with 0.3% TritonX in PBS. Primary antibody was then added and the cultures were incubated further at 4 °C overnight. After 3 more PBS washing steps, secondary antibody and the counterstaining solution were added and incubation at 37 °C was resumed as before (30 min for the 2D systems and 1 h for the 3D ones). Prior to image acquisition, the samples were washed with PBS another 3 times and then stored in PBS at 4 °C.

### 2.6. Enzyme-Linked Immunosorbent Assay (ELISA)

For the 2D culture conditions, after cell detachment and count, a 5 × 10^3^ cell suspension from each cell condition was seeded in a 24-well plate (CLS3527-100EA; Sigma-Aldrich), in a final medium volume of 200 µL/well. The plates were incubated at 37 °C, 5% CO_2_, in humidified atmosphere for 24 h to allow cell attachment. All cell conditions were exposed to silver (Ag) for 24 h. After treatment, the cells were washed twice with warm PBS and the plates were incubated for 3 days at 37 °C, 5% CO_2_, in humidified atmosphere. The samples were collected 3 days later. The 3D spheroids were exposed to Ag for 72 h and incubated in similar conditions for 5 days before collection.

To study the risk of fibrosis in both 2D and 3D constructs, we evaluated the TGFβ expression in the cell supernatant, fractions 1 and 3, using sandwich ELISA kits (TGFβ1-ab100647, Abcam; TGFβ3-LS-F2825, LSBio, Seattle, WA, USA). Before proceeding with the ELISA protocol, the samples were activated with 1 N HCl and 1.2 N NaOH/0.5 M HEPES, while maintaining a pH between 7.0 and 7.6. In addition, the anti-inflammatory IL-10 expression in the cell supernatant was assessed using sandwich ELISA kits (IL-6-ab46042, Abcam).

For each condition, the samples were evaluated in duplicates. The optical density (O.D.) was established by reading the plates at 450 nm using SpectraMax i3 (Molecular Devices, San Jose, CA, USA) for data collection. All samples, reagents, and standards were processed as per manufacturers’ instructions and user manuals.

### 2.7. Quantitative Real-Time PCR (qRT-PCR)

The cell conditions and silver dressing exposure times described in Section 2.6 also apply to our assessment of gene expression for TGF-β1, TGF-β3, IL-6 proteins using quantitative real-time PCR (qRT-PCR). This was done in triplicates for all cell conditions and systems. A minimum of 20 spheroids were required to obtain adequate quantities of RNA. The spheroids were spun down two times for 10 min at 10,000 rpm.

RNA extraction was done with a Rneasy Plus Mini Kit (74134, Qiagen, Venlo, The Netherlands). To obtain the correct dilutions for qRT-PCR, the quantity and purity of RNA were assessed using a NanoDrop 2000/2000c spectrophotometer (ThermoFisher), measured at A260/280 nm and A260/230 nm.

We used primers TGF-β1 (ThermoFisher Hs00998133_m1/4331182), β3 (ThermoFisher Hs01086000_m1/4331182), human IL-6 (ThermoFisher Hs00174131_m1/4331182) and housekeeping gene GADPH (ThermoFisher Hs02786624_g1/4331182). Sample triplicates were prepared using a QuantiTec kit (204443, Qiagen). Gene amplification was done using a Light cycler 480 system (Basel, Switzerland) and gene expression was calculated using the Delta-Delta Ct Method.

### 2.8. Image Acquisition and Assessment

Fluorescent microscopy was performed on a Leica CM6800 microscope (Wetzlar, Germany) with green, red, far-red, and blue cube filters, using 5× and 10× objectives. Confocal imaging was acquired on a CV7000 confocal microscope (Tokyo, Japan). The images were analyzed with the open-source software package Fiji ImageJ (version 1.51s) to assess Integrated Fluorescent Density (IFD) and other parameters, including morphological in the case of 3D images (i.e., area, perimeter, Feret diameter) [35,36].

### 2.9. Statistical Analysis

Statistical analysis was conducted in GraphPad Prism (version 7.00 for Windows) and all reported data are expressed as mean ± SEM (standard error of mean). Monoculture data from treated and untreated conditions were compared using unpaired student’s *t*-test. For cocultures, treated and untreated cell conditions (A20N80 and A80N20) were compared using one-way ANOVA with Sidak’s multiple comparisons test. The significance threshold was set at *p* < 0.05 (95% confidence interval).

The results from the ELISA were calculated as mean absorbance for each set of duplicates, and the negative control optical density was subtracted. The standard curve was generated by plotting the optical density (OD) of serial diluted standards and corresponding known levels. Sample data were interpolated from the standard curve and protein concentrations calculated. Gene expression was assessed using the Delta-Delta Ct Method developed by Livak and Schmittgen [37].

## 3. Results

### 3.1. 2D Culture Systems

#### 3.1.1. Wound Healing Assay

**Figure 3:**
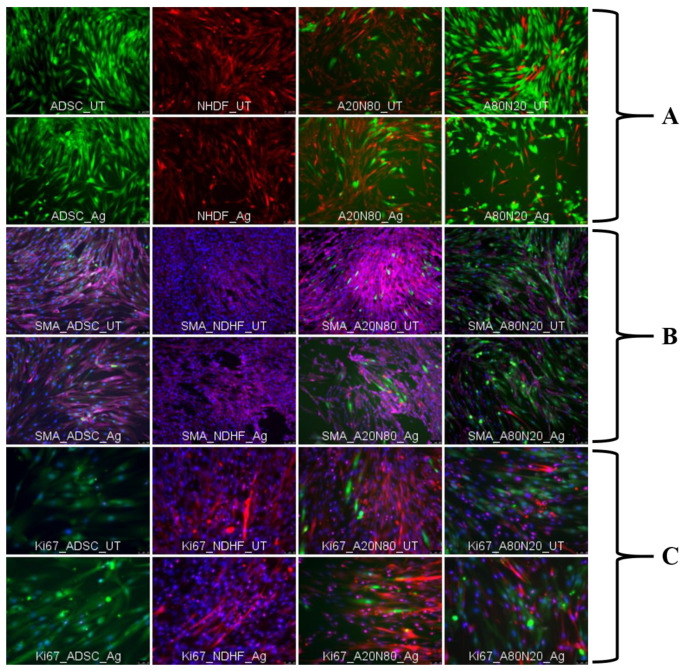
Cultures—(**A**). the gap was not closed in A80N20; (**B**). α-SMA expression (magenta; by convention) with Hoechst nuclear counterstaining (blue), 5 days post-treatment-expression in treated stem cell monocultures was lower compared to untreated; (**C**). Ki-67 expression (magenta; by convention) with Hoechst nuclear counterstaining (blue), 5 days post-treatment (UT—untreated; green—ADSCs; red—NHDFs; blue—Hoechst nuclear counterstaining; magenta—α-SMA/Ki-67) Scale bar: (**A**)—100 µm, (**B**)—100 µm, (**C**)—50 µm.

Following the removal of wound-inserts from their culture dish, all cell cultures were exposed to the silver-coated dressing fragment for 24 h. The cultures were evaluated after another 24 h by fluorescence microscopy (Figure 3A). The wound in the cell monolayer was closed after 24 h in all constructs except the A80N20 cultures, where wound healing was significantly delayed (i.e., gap closure time). On examining migration patterns during the healing stage, stem cell monocultures appeared to engage in single cell movement (mesenchymal migration), with elongated spindle-like cell bodies filling the gap with a cell sheet. The fibroblasts exhibited this mesenchymal migration characteristic as well, except they tended to bridge the gap edges, arranged in a cellular cohort, similar to a collective migration type. The same migration pattern was observed in the A20N80 condition, but the stem cells in those cultures lost their spindle-like shape and were surrounded by fibroblasts in a cellular cohort. The findings were similar for both treated and untreated cultures except for A80N20. For conditions with dominant stem cell fraction (80%), following silver dressing treatment, the stem cells adopted a more polygonal shape instead of a spindle-like shape, losing their multipolar elongated phenotype. Furthermore, their migration pattern was an individual cell event unable to close the wound after 24 h.

**Figure 4:**
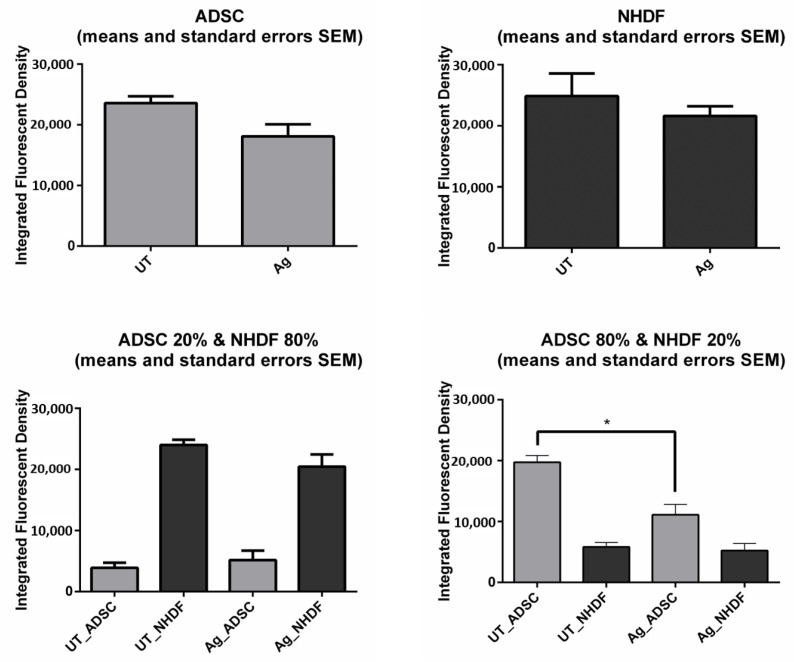
2D wound healing assay—Integrated Fluorescent Density (IFD) measurements (means with SEM): Ag exerted cytotoxicity on stem cells in ADSC monocultures (*p* < 0.05 *, unpaired *t*-test) and A80N20 cocultures (one-way ANOVA test).

The comparative statistical analysis of IFD image data for untreated versus treated monocultures and cocultures is summarized in Figure 4. For monocultured fibroblasts, mean IFD values were 24,855 ± 1510 units versus 21,618 ± 1575, respectively, with non-significant differences indicating no meaningful changes in cell number upon exposure to silver (*p* = 0.271; n = 6). Similarly, only minimal statistical differences were observed between untreated vs. treated stem cells (*p* = 0.0371). For cocultures, a significant statistical difference was noted in the A80N20 cocultures rich in stem cells, indicating a strong cytotoxic effect to the stem cell population (*p* < 0.05), while the effect on fibroblasts was non-significant.

#### 3.1.2. Protein Expression in 2D Cultures—Immunofluorescent Assay

**Figure 5:**
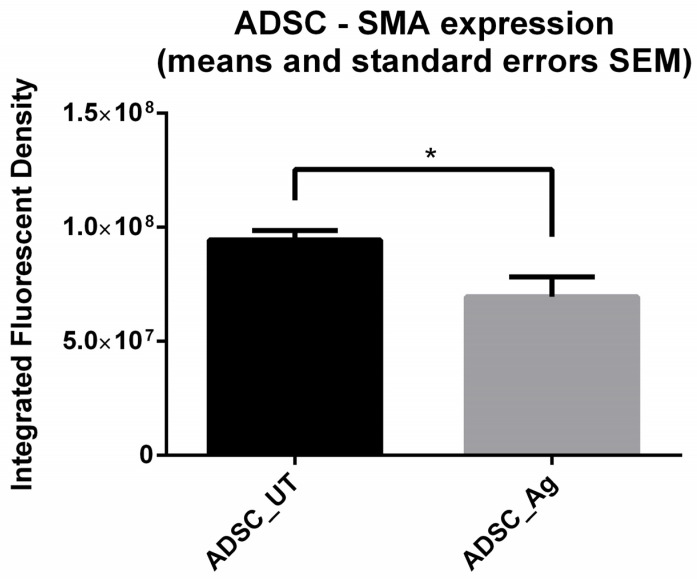
Integrated Fluorescent Density (IFD) measurements (means with SEM) of α-SMA protein expression in 2D ADSC monocultures: α-SMA protein expression increased in treated versus untreated cultures (*p* < 0.05 *).

Expression of α-SMA protein in stem cell monoculture seems to be modulated following treatment (Figure 3B). When IFD is assessed, there is a highly significant difference (*p* < 0.05) between the expression in native ADSC and after exposure to Ag (Figure 5). In cocultures, stem cells seem to express less α-SMA, and they are surrounded by fibroblast expressing high quantities of α-SMA, both native and post-treatment. It seems that α-SMA expression in both cell types is modulated in the presence of fibroblasts in a coculture environment.

In the monocultures, both stem cells and fibroblasts expressed the proliferation marker Ki-67 regardless of treatment (Figure 3C). The stem cells in the untreated A20N80 samples did not express Ki-67 and they were surrounded by fibroblasts which did express this proliferation marker. In the corresponding samples exposed to silver, a fraction of the stem cells expressed Ki-67. By contrast, both cell types in A80N20 monocultures expressed Ki-67 whether or not treated with silver.

### 3.2. 3D Culture Systems

#### 3.2.1. Sprouting Assay

**Figure 6:**
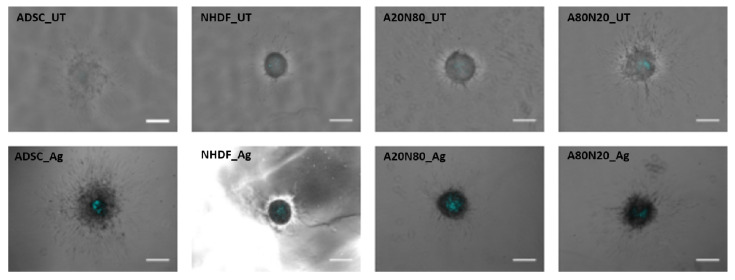
Viability assessment with SytoxBlue: all cell constructs maintained viability following sprouting assay (UT—untreated; blue—SytoxBlue; scale bar—250 µm).

**Figure 7:**
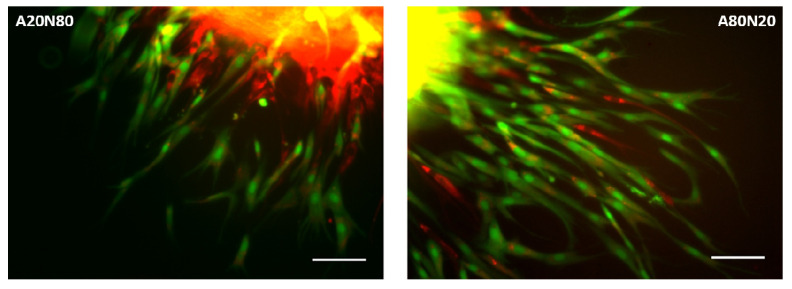
Confocal images representative of observed behavior in untreated cocultures A20N80 and A80N20 [38]: stem cells and fibroblasts in both constructs featured sprouting spikes, especially in A80N20 (green—ADSCs; red—NHDFs; yellow shades due to overlay; scale bar—100 µm).

First, all four constructs, in both treated and untreated conditions, maintained viability following sprouting assay, as shown in Figure 6. Additionally, the confocal image acquisition and evaluation of cell distribution in untreated cocultures is illustrated in Figure 7.

**Figure 8:**
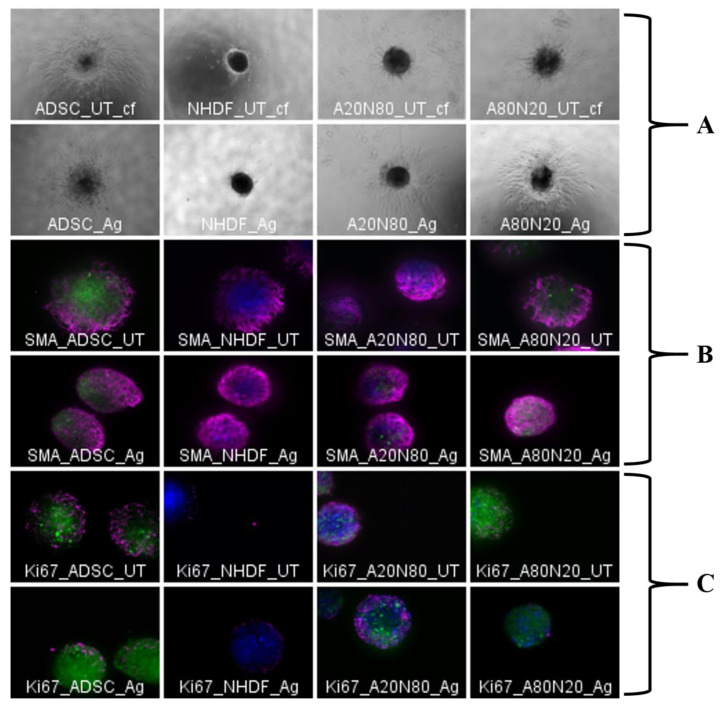
3D cultures, 10×: (**A**). Spheroid sprouting assay, 3 days post-treatment, bright field; (**B**). α-SMA expression (magenta; by convention) with Hoechst nuclear counterstaining (blue), 5 days post-treatment; (**C**). Ki-67 expression (magenta; by convention) with Hoechst nuclear counterstaining (blue), 5 days post-treatment (UT—untreated; green—ADSCs; red—NHDFs; blue—Hoechst nuclear counterstaining; magenta—Ki-67).

Spheroids were assessed 3 days after collagen droplet embedding. Images were evaluated from migration pattern point of view and measurements were performed by ImageJ (Figure 8A). Fluorescence imaging was not possible due to debris from dressing fragments. The untreated stem cell monocultures displayed a well-defined proliferation zone with astral outgrowth. At the periphery of the sprouting zone, the cells started to detach from the spheroid. Following treatment, the spheroid core contract areas, perimeters, and Feret diameters were smaller compared to untreated samples, and branching was less robust.

Prior to treatment, the fibroblast monocultures showed less propensity toward the formation of spikes; migrated cells tended to detach from the spheroid, while the core remained compact. Following exposure to silver, spikes forming the spheroids were less well-defined, although the cells continued detaching.

The A20N80 coculture constructs exhibited a similar migration pattern prior and post-treatment, with the core morphological parameters after exposure resembling the fibroblast-only spheroids. For constructs with dominant stem cells fraction (A80N20), a higher migration rate was observed, and spheroids developed a significant proliferation zone and well-defined spikes.

#### 3.2.2. Protein Expression in 3D Cultures—Immunofluorescent Assay

Immunofluorescence images were assessed semi-quantitatively, including morphologically (area, perimeter, and Feret diameter). All cell constructs expressed native α-SMA, and protein expression appeared to increase in all four cell conditions after exposure to silver for 3 days (Figure 8B). General and morphological parameters indicated spheroid contraction after silver exposure. To assess morphological changes following treatment, we normalized the Raw Integrated Density measured for the far-red fluoresce channel (for the secondary antibody Fluo647) with the spheroid projection area, measured manually. The results were consistent with the qualitative assessment of the α-SMA from the images and demonstrate the increase in Raw Integrated Density corresponding to the increase in the area of the spheroid projections following silver treatment.

The proliferation marker was assessed qualitatively only, due to its intra-nuclear expression and image overlays (Figure 8C). For the stem cells monocultures and the coculture construct with dominant stem cells fraction, Ki-67 expression seems to diminish after treatment. The proliferation marker in the fibroblast monocultures increased slightly after exposure to silver. Furthermore, when fibroblasts were cocultured with 20% stem cells, the construct’s response to treatment was similar to that of the untreated counterpart.

### 3.3. Pro/Anti-Fibrosis Markers—Levels and Gene Expression

**Figure 9:**
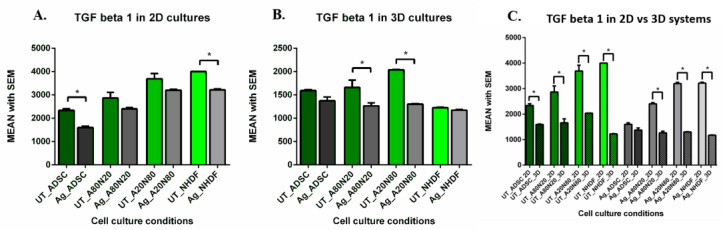
TGFβ-1 levels (pg/mL): (**A**). 2D systems—TGFβ-1 decreased post-treatment, significantly more in treated vs. untreated ADSC and NHDF (* *p* < 0.0001); (**B**). 3D systems—TGFβ-1 decreased post-treatment in A80N20 and A20N80 (* *p* < 0.0002); (**C**). TGFβ-1 in 2D vs. 3D systems: TGFβ-1 decreased in both untreated and treated conditions (* *p* < 0.0001), except for treated ADSC (non-significant decrease).

In the 2D systems, 3 days after 24 h-exposure to silver, TGFβ-1 levels in the cell supernatant varied significantly depending both on treatment and cell condition (Figure 9A). Although TGFβ-1 levels decreased post-treatment across all studied conditions, we noted a strongly significant statistical difference between treated and untreated monocultures (*p* < 0.0001). Furthermore, TGFβ-1 levels were significantly lower for treated ADSC monocultures compared with NHDF monocultures (*p* < 0.0001). When treated cell conditions were compared, a pattern emerged whereby TGFβ-1 levels decreased as the ADSC culture fraction increased, and differences were significant between ADSC vs. A20N80, ADSC vs. A80N20, A80N20 vs. A20N80 and A80N20 vs. NHDF. Concurrently, untreated and treated cocultures with fibroblasts as a majority fraction did not present significant differences.

In the 3D systems, although TGFβ-1 levels decreased after Ag exposure across all cell conditions, differences were significant only between treated and untreated coculture conditions (*p* < 0.0002) (Figure 9B). When comparing same cell and treatment conditions, TGFβ-1 levels decreased more substantially in all cell conditions cultured in 3D systems relative to 2D systems (*p* < 0.0001), except for treated ADSC monocultures (Figure 9C).

**Figure 10:**
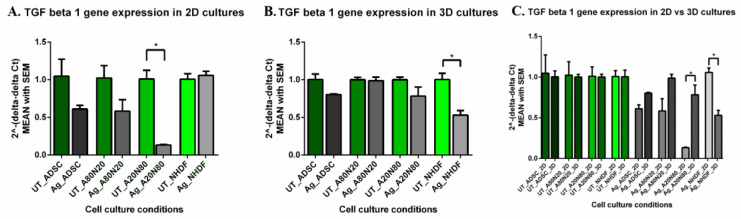
TGFβ-1 gene expression: (**A**). 2D systems—post-treatment down-regulation in ADSC mono and cocultures, with significant difference for A20N80 (* *p* < 0.0005); (**B**). 3D systems—post-treatment down-regulation in NHDF (* *p* < 0.0007); (**C**). TGFβ-1 gene expression in 2D vs. 3D culture systems: minimal changes in untreated cultures; post-treatment, up-regulation in ADSC mono and cocultures, and down-regulation in NHDF in 3D systems (* *p* < 0.0001).

TGFβ-1 gene expression in the 2D systems was down-regulated after Ag exposure in ADSC monocultures and cocultures, especially in the A20N80 cocultures (*p* < 0.0005). In NHDF monocultures, although we saw a minimal increase in TGFβ-1 gene expression, there were no significant differences between treated and untreated conditions. Furthermore, when treated cell conditions were compared, TGFβ-1 gene expression was significantly down-regulated in treated A20N80 compared with treated NHDF monocultures (*p* < 0.0005) (Figure 10A).

In the 3D systems, treatment appeared to down-regulate TGFβ-1 gene expression in ADSC monocultures and A20N80, although non-significantly. In NHDF monocultures, on the other hand, TGFβ-1 gene expression was down-regulated significantly after Ag exposure (*p* < 0.0007). Moreover, between A80N20 and NHDF monocultures, we found TGFβ-1 gene expression decreased significantly in treated fibroblast monocultures (*p* < 0.0007) (Figure 10B).

Comparing the 2D and 3D systems, there were minimal shifts in TGFβ-1 gene expression across all untreated conditions. TGFβ-1 gene expression was up-regulated in 3D systems of treated ADSC mono and cocultures, with a significant difference between treated A20N80 in 2D vs. 3D cultures. In treated NHDF 3D monocultures, TGFβ-1 gene expression was significantly down-regulated compared to the corresponding treated 2D cultures (*p* < 0.0001) (Figure 10C).

**Figure 11:**
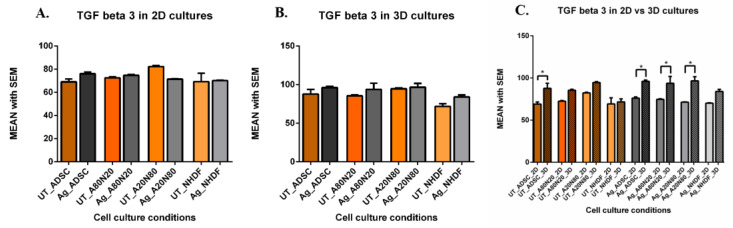
TGFβ-3 levels (pg/mL): (**A**). 2D systems—non-significant post-treatment increases in ADSC and A20N80; (**B**). 3D systems—minimal increases post-treatment; (**C**). TGFβ-3 levels (pg/mL) in 2D vs. 3D culture systems: increased levels in both untreated and treated 3D vs. 2D systems, with significant differences for untreated ADSC and for treated ADSC mono and cocultures (* *p* < 0.0001).

Although TGFβ-3 levels generally increased after Ag exposure in the case of the 2D ADSC monocultures and A80N20 cocultures, these changes were not significant. Likewise, there was no major shift between untreated and treated NHDF monocultures in A20N80 cocultures, even if the level of TGFβ-3 decreased after Ag treatment (Figure 11A). In the 3D systems, TGFβ-3 levels increased slightly after Ag treatment of the ADSC, A80N20 and NHDF cultures, while levels remained similar in treated vs. untreated cultures in A20N80 cocultures (Figure 11B). The TGFβ-3 level increased in all untreated 3D conditions compared to 2D, yet statistically significant only in the case of ADSC monocultures (*p* < 0.0001). Furthermore, TGFβ-3 levels increased significantly in 3D systems of ADSC mono and cocultures after Ag exposure compared with their 2D counterparts (*p* < 0.0001) (Figure 11C).

**Figure 12:**
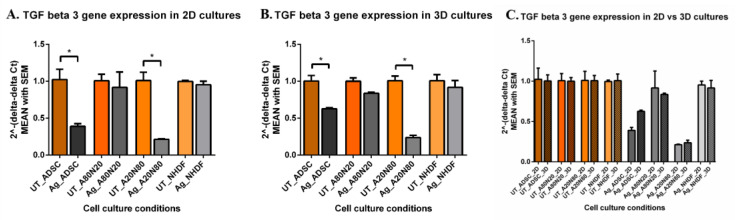
TGFβ-3 gene expression: (**A**). 2D systems—down-regulation in treated ADSC and A20N80 (* *p* < 0.0001); (**B**). 3D systems—down-regulation, mainly in treated ADSC and A20N80 (* *p* < 0.0001); (**C**). TGFβ-3 gene expression in 2D vs. 3D culture systems: minimal changes in untreated conditions; up-regulation in ADSC and A80N20, and down-regulation in 80N20 and NHDF (non-significant).

In the 2D systems, TGFβ-3 gene expression was down-regulated after Ag exposure, significantly in the case of ADSC monocultures and A20N80 cocultures. Gene down-regulation was more significant for treated ADSC and A20N80 cultures when compared to treated NHDF and A80N20 cultures (*p* < 0.0001) (Figure 12A). In the 3D systems, TGFβ-3 gene expression was down-regulated in all treated cell conditions compared to untreated ones, with significant differences for ADSC monoculture and A20N80 cocultures (*p* < 0.0001). When comparing treated conditions, TGFβ-3 gene expression was significantly more down-regulated for A20N80 cultures relative to NHDF and A80N20 cultures, and between ADSC and NHDF monocultures (Figure 12B). TGFβ-3 gene expression did not differ significantly between 2D and 3D systems, with comparable levels observed under untreated conditions. TGFβ-3 gene expression was up-regulated in treated ADSC cultures and A20N80 cocultures and down-regulated for treated NHDF and A80N20 cultures in 3D systems compared with 2D systems (Figure 12C).

### 3.4. Pro/Anti-Inflammation Markers—Levels and Gene Expression

**Figure 13:**
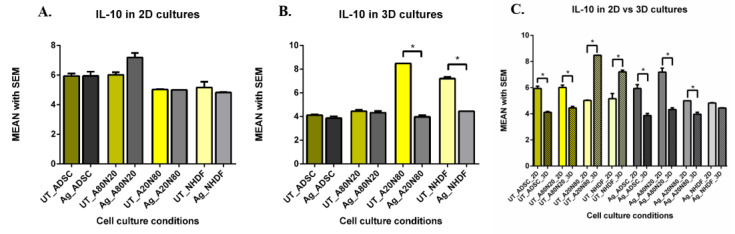
IL-10 levels (pg/mL): (**A**). 2D systems—post-treatment IL-10 increased in A80N20 versus ADSC, A20N80, and NHDF; (**B**). 3D systems—post-treatment IL-10 decreased in A20N80 and NHDF (* *p* < 0.0001); (**C**). IL-10 levels (pg/mL) in 2D vs. 3D systems: IL-10 decreased in untreated 3D ADSC and A80N20, and increased in treated A20N80 and NHDF compared to untreated 2D counterparts; IL-10 decreased in treated 3D versus 2D systems, significantly for ADSC, A20N80, and A80N20 (* *p* < 0.0001).

The levels of IL-10 increased non-significantly after Ag exposure in the 2D A80N20 cocultures compared with untreated counterparts, and increased significantly for treated A80N20 cocultures compared with all other treated cell conditions (*p* < 0.0009) (Figure 13A). In the 3D systems, IL-10 levels decreased significantly for treated A20N80 cocultures and NHDF monocultures compared with untreated conditions (*p* < 0.0001) (Figure 13B). For untreated conditions, we found significant reductions in IL-10 levels in the ADSC monocultures and A80N20 cocultures, while in 3D NHDF monocultures and A20N80 cocultures the IL-10 levels increased significantly compared with same cell conditions in 2D systems (*p* < 0.0001). Concurrently, significantly lower levels of IL-10 were recorded in the ADSC, A20N80, and A80N20 cultures in the 3D versus 2D systems (*p* < 0.0001) (Figure 13C).

**Figure 14:**
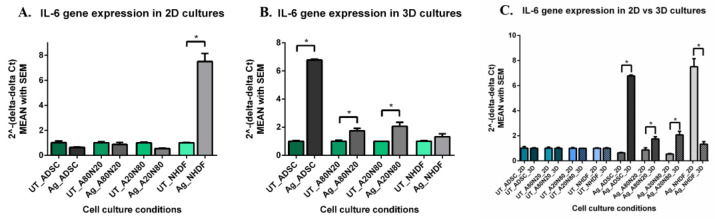
IL-6 gene expression: (**A**). 2D systems—higher up-regulation in treated NHDF vs. untreated NHDF and other treated constructs (* *p* < 0.0001); (**B**). 3D systems—up-regulation in all treated conditions, especially treated ASDC mono- and cocultures (* *p* < 0.0001); (**C**). IL-10 gene expression in 2D vs. 3D culture systems: similar expression in untreated cultures; in treated 3D systems, expression was up-regulated in ADSC mono and cocultures, and down-regulated in NHDF (* *p* < 0.0001).

IL-6 gene expression was slightly and non-significantly down-regulated in the treated ADSC monocultures and A20N80 cocultures from the 2D systems. In the case of treated NHDF monocultures, on the other hand, IL-6 gene expression was strongly up-regulated compared to both untreated counterparts and the other treated cell constructs (*p* < 0.0001) (Figure 14A). In all treated 3D systems, IL-6 gene expression was up-regulated, with significant differences noted between treated and untreated ADSC, A20N80, and A80N20 constructs. In fact, IL-6 gene expression was significantly up-regulated in all the other treated cell constructs and more substantial in the A20N80 cocultures relative to the NHDF monocultures (*p* < 0.0001) (Figure 14B). At the same time, IL-6 gene expression was relatively similar across the untreated cultures from both 2D and 3D systems. When comparing 3D with 2D systems after Ag exposure, we found significant up-regulation of the IL-6 gene expression in ADSC mono and cocultures, and down-regulation in the NHDF monocultures (*p* < 0.0001) (Figure 14C).

## 4. Discussion

In wound healing research, silver-coated dressings have been assessed mainly using 2D monocultures with fibroblasts and keratinocytes, or in vivo [23,24,28,39,40]. In our experimental design, we used a range of 2D and 3D cell culture systems with fibroblasts and adipose-derived stem cells, both known to play important roles in wound healing, and assessed the effects of silver treatment.

The impact of silver on wound healing processes is yet to be fully elucidated. For instance, Poon et al. demonstrated in vitro the cytotoxicity effect of silver on fibroblasts [39]. Burd et al. evaluated, also in vitro, the effects of silver on fibroblasts and keratinocytes, and demonstrated in vivo delayed healing [24]. Conversely, Wright et al. later used an experimental pig model to show that silver improved wound healing in the early phases in the in vivo study [25]. Furthermore, Hiro et al. compared in vitro and in vivo effects and, while in vitro silver triggered cytotoxicity, in vivo silver treatment appeared to accelerate healing [41]. Current data, therefore, presents a discrepancy between the two model systems which justifies further research and discussion.

In our observations, the wound healing assay on 2D systems revealed nuanced differences in impact, such as depending on the cell population ratio between stem cells and fibroblasts. Even if the wound gap closed after 24 h in both monocultures and under both treated and untreated scenarios, silver treatment was found to exert a significant cytotoxic effect on stem cells (*p* < 0.05 *, R-squared 0.81) measured as IFD quantified from images. At the same time, in coculture systems with dominant ADSC (A80N20), the gap did not close after 24 h and the IFD was significant lower following treatment (*p* < 0.05 *), indicating a negative effect of silver treatment on stem cell migration and, by consequence, wound healing potential. This aligns with the findings of Hackenberg et al., who demonstrated cyto- and genotoxic effects of silver nanoparticles at different concentrations [26]. In addition, Perez-Diaz et al. used ADSC and silver nanoparticles in a biomatrix to develop a nanocomposite for skin wound management. Although the stem cells displayed a 35% reduction in cell viability, the construct was permissive for cell proliferation [27].

Cellular α-SMA expression plays an important role in cell migration and migration patterns [32]. In our monocultures, although the ADSCs displayed significantly reduced α-SMA expression following silver treatment (*p* < 0.05 *), suggesting negative modulation of stem cell migration/mobility, the fibroblasts did not undergo significant change post-treatment; the fact that the gap closed after 24 h for both cell types suggests that closure may have been driven primarily by the fibroblast cell population. Furthermore, the presence of fibroblasts in cocultures seems to have modulated shifts in protein expression regardless of the cell type ratio, although ours were IFD calculations were global and not per cell type. Even if we found no significant alteration in α-SMA expression in either coculture, the gap was not closed after 24 h in the A80N20 cultures, suggesting that α-SMA presence does not correlate with cell migration potential. Suppression of α-SMA following treatment may be significant in clinical settings by reducing the risk of excessive scar tissue development, as high levels of α-SMA have been associated with hypertrophic scars [42,43]. Further study weighing the benefits of silver-coated dressing usage against such risks is necessary. Hypertrophic scars develop especially during the remodeling phase of the wound healing process, usually by the 3rd to 4th weeks. At this stage, in a normal scar, the rich α-SMA fibroblasts would undergo apoptosis, while in a hypertrophic scar tissue they become resistant and contribute to the formation of fibrous scar tissue [43].

Protein Ki-67 is routinely used as an active proliferation marker, based on its presence throughout the active phases of the cell cycle and absence in resting cells (G0) [44]. In both monocultures and A80N20 cocultures, Ki-67 marker expression seemed unaffected by the silver treatment. In the treated A20N80 cocultures, although the stem cells remained in a quiescent state, defined by lack of Ki-67, a fraction of ADSC samples started to express Ki-67 following exposure to silver, evidence of initiation of the active phase in the cell cycle.

Under physiological conditions, different cell types come into contact with each other, and their phenotypes and movements are influenced by the three-dimensional organotypic environments they operate in; this is a characteristic that 2D culture systems cannot mimic. It has also been widely observed that empirical data obtained from 3D culture models often provide better correlation with physiological/in vivo settings and higher translation rates of research findings into clinical practice [28,45]. A 2D culture comprises a monolayer of proliferating cells equally exposed to the controlled media and treatment conditions, whereas a 3D culture features a heterogeneous mixture of cells in different stages (e.g., proliferating, quiescent, necrotic) which get exposed differently to the given media and treatment conditions, with a diffusion gradient from the necrotic core toward the proliferating zone. At the same time, 2D cell cultures are still common practice due to the simple, time-saving, and low-cost setup. To provide comprehensive assessment and overcome the inherent limitations of either methodology, we designed the study to include both 2D and 3D approaches, and we also varied exposure times to the silver-coated dressing between these constructs.

According to a study by Rigo et al., who evaluated 3D fibroblast monocultures from biopsy samples of partial thickness burns from patients treated with silver dressings, the fibroblasts maintained viability and nuclear membrane integrity, although mitochondrial activity declined. Their in vitro data did not reveal any wound healing impairments or viability issues during silver treatment [29]. Our data on the 3D culture constructs is consistent with such findings, suggesting that silver does not impede wound healing. We also documented no significant changes in the sprouting pattern between treated and untreated conditions of all 3D cultures. Furthermore, while cells in the A80N20 cocultures did not close the wound gap by the 24 h mark in the wound healing assay, in contrast, the proliferation zone in the 3D constructs was well developed, with clearly defined spikes projected outward, indicating high migration/regeneration potential, unlike in the 2D culture systems.

Interestingly, the α-SMA protein expression increased post-treatment in all 3D cell culture conditions compared to the 2D systems, where protein expression was lowered. This contradicting result requires further investigation, as this protein bears some significance in scar development, even if biological evidence connecting silver usage and the risk for hypertrophic scar development is currently limited.

TGFβ isoforms are multifunctional cytokines which modulate a range of different processes important in wound healing events, such as cell migration/differentiation, extracellular matrix synthesis, and epithelial–mesenchymal interplay, possibly also stem cell recruitment and differentiation. Their reported effects in vitro have been consistent; however, in vivo, TGFβ isoforms have been found to display functional and expression variations, mainly related to microenvironment conditions. Data regarding the impact of TGFβ-1 on stem cell proliferation specifically is sparse, but early findings suggest a bi-phasic effect dependent on level [5,6,7,46,47,48,49,50].

Moreover, TGFβ-1 plays an important role in healing by modulating fibroblast activity, myofibroblast conversion, and fibrous tissue formation, while its overexpression is associated with fibrosis [10,51,52]. TGFβ-3 is also associated with fetal wound healing phenotype and is considered an anti-fibrotic factor [13,51,53]. Currently, the interplay between TGFβ and ADSCs has been documented but not fully explored. On one hand, ADSCs may inhibit TGFβ-1 expression, according to some studies; in turn, ADSCs exposed to high amounts of TGFβ-1 have been found to convert to myofibroblast-like phenotype, with increased αSMA expression through Smad2 pathway activation [54,55]. You et al. designed an in vivo study to evaluate the effects of a scaffold loaded with silver nanoparticles on different growth factors and interleukins. They demonstrated that local levels of IL-6 and TGFβ decrease, with IL-10 amounts increasing after silver exposure, suggesting a beneficial anti-inflammatory effect on would healing outcomes [30]. Herein, TGFβ-1 levels in the studied 2D monoculture systems declined following exposure to Ag, and significant post-treatment decrease was also noted in the 3D coculture models. We can therefore confirm the literature data that Ag inhibits TGFβ-1 and, as such, may have an anti-inflammatory effect. Still, the measured levels seem to have also been modulated by the cell types in the constructs and culture models. Although no statistically significant differences in TGFβ-3 were recorded, levels increased after Ag exposure, mainly in ADSC monocultures and the cocultures having stem cells as a majority fraction.

Additionally, it is well-known that TGFβ-1 is a crucial factor in triggering myofibroblast-like phenotype in mesenchymal cells, with subsequent increase in α-SMA. In our treated 3D experimental setup, TGFβ-1 levels decreased while α-SMA expression increased, which suggests other factors may have been involved in α-SMA modulation. Desai et al. evaluated myofibroblast activity using human ADSCs treated with conditioning media containing either TGFβ-1 or basic fibroblast growth factor. They reported decreasing α-SMA when ADSCs were exposed to high levels of basic fibroblast growth factor, and the opposite when exposed to TGFβ-1, suggesting that myofibroblast differentiation could be modulated by various growth factors. Moreover, the ADSC phenotype switched from one profile to the other depending on the growth factor used, indicating that myofibroblast differentiation is not an end-point process, and reiterating stem cell plasticity [55]. Thus, TGFβ-1 is the leading known growth factor in myofibroblast-like phenotype, while other growth factors seem to be involved as well. Further research is required regarding α-SMA modulation in various scenarios (e.g., cell culture systems, treatments) by including other growth factors such as basic fibroblast growth factor.

IL-10 is believed to play a role in fetal wound healing phenotype by modulating the inflammatory response; IL-10 inhibits pro-inflammatory cytokines, including IL-6, and inflammatory cell migration. Low IL-10 levels are associated with scar and fibrous tissue formation [4,13,14]. In our 2D cell models, IL-10 levels in the A80N20 cocultures increased slightly following Ag exposure; in the 3D cell models, IL-10 levels decreased, significantly more so in the NHDF monocultures and cocultures with fibroblasts as a majority fraction. This, again, suggests that the Ag effects differ depending on cell types and culture systems.

IL-6 is a cytokine with a well-known pro-inflammatory primary role despite evidence of anti-inflammatory and regenerative effects, depending on the activated pathway. Abnormalities in IL-6 secretion have been associated with impaired wound healing [3,10,11,12]. In the present study, IL-6 gene expression was up-regulated after Ag exposure of 3D systems, mainly in the ADSC mono- and cocultures. These results suggest that both cell population and exposure to silver-based dressing modulate IL-6 gene expression, although the signaling pathway was not investigated. Hackenberg at al. evaluated the effects of silver nanoparticles on stem cells, showing an in vitro cytotoxic effect associated with DNA mutations, and significant IL-6 increase following silver exposure [26].

With regard to the effects of antiseptics on wound healing and scarring, current data are controversial and difficult to translate to clinical setups. Pathological scaring is specific to humans and in vivo models are limited [8]. Although relevant, in vitro studies, mainly on 2D culture systems, have significant drawbacks. As explained previously, 3D cultures provide a microenvironment that mimics physiological conditions better, with higher predictability for in vivo conditions. However, experiments using 3D culture setups have yielded different results, mainly due to the spatial display possibilities of the cell types and gene expression modulation. In 2D setups, the cells are laid out in a monolayer, thus displaying different morphology; as a result, they are exposed uniformly to media and treatment, achieving higher proliferation rates compared to in vivo settings. Thus, generated data may be unreliable regarding cytotoxicity and clinical translation [28,39]. In this study, we found significant differences in pro/anti-inflammatory and pro/anti-fibrotic markers modulation following Ag exposure between 2D and 3D systems. Lower TGFβ-1 and higher TGFβ-3 levels were more readily observable in 3D versus 2D systems, suggesting a post-treatment anti-inflammatory response mainly for ADSC cocultures. For ADSC mono and cocultures, IL-10 levels and IL-6 gene expression significantly shifted after treatment in 3D versus 2D cell models.

The main limitation in this study is related to image data collection and quantification for fluorescence, as fluorescence microscopy requires image channels overlaying which proved a major obstacle with open-source image processing tools. Additionally, overlaying is more pronounced and difficult to manage in 3D culture systems due to the spheroid thickness and imaging limitations, which is why our study was semi-qualitative. We also did not evaluate how different cells/cell types were spatially arranged and how they interacted within the spheroid structures, or any phenotype changes associated with the adipose-derived stem cells in the cocultures.

Another limitation is that the pluripotency and differentiation ability was not evaluated before or after treatment. The available literature data suggests that silver nanoparticles do not impact ADSC differentiation potential, and that the pluripotency of stem cells increases in 3D culture systems relative to monolayered 2D cultures [56,57].

Finally, regarding the studied pro/anti-fibrotic and pro/anti-inflammation markers, it is worth noting that the experimental treatment exposure conditions and subsequent times were different in the 2D and 3D systems, and all assessments were performed as non-dynamic end-points. For Il-6, specifically, only gene expression was evaluated, without pathway signaling or post-translational analysis, so up- or down-regulations cannot be correlated with anti- or pro-inflammatory effects. These limitations are a call for further research to elucidate the exact role of IL-6 gene expression modulation following cell exposure to silver-based dressing, and the downstream effects, mainly exploring the STAT3 signaling pathway. This is important especially as TGFβ modulation was observed alongside IL-6 gene expression, and there is available data on how both TGFβ and IL-6 can activate the STAT3 pathway with impact on wound healing and scar tissue formation [58].

## 5. Conclusions

Silver-based dressings may exert anti-inflammatory and anti-fibrotic effects, especially when additional adipose-derived stem cells are used; thus, simultaneous usage of stem cell-based therapy and silver dressings may be beneficial for wound healing. The influence of ADSCs and silver-based dressing on IL-6 levels requires further investigation, mainly on the two different signaling pathways.

Although silver appeared to have a cytotoxic effect under 2D culture systems, this was non-significant, and results from the physiologically relevant 3D culture models suggest overall beneficial effects of silver on wound healing. As 3D culture systems, especially organotypic cultures, can mimic physiological environments more closely, it would be appropriate to expand the use of 3D culture/coculture systems as study tools to generate in vitro data with greater potential to translate into clinical applications and outcomes.

## Data Availability

All data of this study are included within the article.

## Abbreviations

The following abbreviations are used in this manuscript:

α-SMA	α-smooth muscle actin
A20N80	Coculture with 20% adipose-derived stem cells and 80% fibroblasts
A80N20	Coculture with 80% adipose-derived stem cells and 20% fibroblasts
ADSC	Adipose-derived stem cells
Ag	Silver-coated dressing
ELISA	Enzyme-linked immunosorbent assay
IFD	Integrated fluorescent density
IL	Interleukin
qRT-PCR	Quantitative real time polymerase chain reaction
NHDF	Normal human dermal fibroblasts
PBS	Dulbecco phosphate-buffered saline
TGFβ	Transforming growth factor beta
UT	Untreated
